# Profiling of Volatile Organic Compounds in Exhaled Breath As a Strategy to Find Early Predictive Signatures of Asthma in Children

**DOI:** 10.1371/journal.pone.0095668

**Published:** 2014-04-21

**Authors:** Agnieszka Smolinska, Ester M. M. Klaassen, Jan W. Dallinga, Kim D. G. van de Kant, Quirijn Jobsis, Edwin J. C. Moonen, Onno C. P. van Schayck, Edward Dompeling, Frederik J. van Schooten

**Affiliations:** 1 Department of Toxicology, Nutrition and Toxicology Research Institute Maastricht (NUTRIM), Maastricht University, Maastricht, The Netherlands; 2 Top Institute Food and Nutrition, Wageningen, The Netherlands; 3 Department of Pediatric Pulmonology, School for Public Health and Primary Care (CAPHRI), Maastricht University Medical Center (MUMC), Maastricht, The Netherlands; 4 Department of General Practice, School for Public Health and Primary Care (CAPHRI), Maastricht University Medical Center (MUMC), Maastricht, The Netherlands; Research Center Borstel, Germany

## Abstract

Wheezing is one of the most common respiratory symptoms in preschool children under six years old. Currently, no tests are available that predict at early stage who will develop asthma and who will be a transient wheezer. Diagnostic tests of asthma are reliable in adults but the same tests are difficult to use in children, because they are invasive and require active cooperation of the patient. A non-invasive alternative is needed for children. Volatile Organic Compounds (VOCs) excreted in breath could yield such non-invasive and patient-friendly diagnostic. The aim of this study was to identify VOCs in the breath of preschool children (inclusion at age 2–4 years) that indicate preclinical asthma. For that purpose we analyzed the total array of exhaled VOCs with Gas Chromatography time of flight Mass Spectrometry of 252 children between 2 and 6 years of age. Breath samples were collected at multiple time points of each child. Each breath-o-gram contained between 300 and 500 VOCs; in total 3256 different compounds were identified across all samples. Using two multivariate methods, Random Forests and dissimilarity Partial Least Squares Discriminant Analysis, we were able to select a set of 17 VOCs which discriminated preschool asthmatic children from transient wheezing children. The correct prediction rate was equal to 80% in an independent test set. These VOCs are related to oxidative stress caused by inflammation in the lungs and consequently lipid peroxidation. In conclusion, we showed that VOCs in the exhaled breath predict the subsequent development of asthma which might guide early treatment.

## Introduction

During the few last decades biomarker discovery has become a significant area in biomedical research. It focused on delivery of diagnostic tools for accurate assessment when a healthy state becomes dysfunctional at the earliest stage possible. The conjunction of advanced spectroscopy with multivariate analysis allows the detailed breakdown of the molecular composition of biofluids, cells and/or tissues [1]. Multivariate analysis enables extraction of the information of interest from numerically large and complex biological data. Breathomics or the analysis of the exhaled breath is used less as a biological medium for metabolomics testing then biofluids [2], [3]. Similarly to other types of biological samples, exhaled breath can be used to define biomarkers (or breath prints) related to abnormal health status in humans. The metabolites detected in exhaled breath originate from normal and deviant (for instance inflammatory) metabolic processes occurring in the body [4], [5]. These processes produce volatile products, Volatile Organic Compounds (VOCs), which are first released into the blood and ultimately in the lungs into exhaled breath. Many degenerative diseases are related to a form of chronic inflammation and/or oxidative stress that leads to the excretion of specific volatile compounds [3], [6]. Several applications of breathomics have been demonstrated for monitoring and diagnosing diseases, such as asthma [7], [8], lung cancer [9], chronic obstructive pulmonary disease [10]–[12], cystic fibrosis [13], [14], inflammatory bowel disease [15] and non-alcoholic steatohepatitis [16].

We propose in this paper to use breathomics for the early diagnosis of asthma. This disease involves inflammatory processes and therefore is a suitable target for breathomics. Asthma is the most common chronic illness in childhood and is defined as a chronic condition with symptoms of wheezing, cough and difficulties in breathing [17], [18]. Though worldwide around 30% of preschool children have at least one asthmatic symptom such as coughing, wheezing and dyspnea [18], only one third will develop asthma at later in life. The remaining group of children will have transient symptoms, also called viral wheeze [19], [20]. Nevertheless, in preschool children, wheeze is a respiratory symptom which is very often a reason for consulting a physician. The etiology of preschool wheeze is complex and its development is a combination between genetic predisposition and environmental factors like allergens, passive smoking, pollution and infections [21].

Diagnosing asthma in preschool children is very challenging. Several tests can support the diagnosis of asthma, such as spirometry, bronchoprovocation and sputum induction [22]. However, these tests are very difficult to perform with preschool children due to their invasiveness and to the necessity of active cooperation [23], [24]. Therefore, it is currently impossible to reliably predict what percentage of wheezing children will develop asthma. Trustworthy diagnosis of asthma is only possible around the age of six [25]. Thus identification of preschool children developing asthma remains a challenge. Early diagnosis of asthma in preschool children might improve specific therapies or justify secondary prevention interventions [26]. The application of breathomics seems well indicated here because of its noninvasive nature and the great potential of applications in disease diagnosis and monitoring [3]. Therefore the aim of the current study is to investigate whether breath VOCs profiles are capable of discriminating preschool children with asthma from transient wheezing early after onset of wheezing. For that purpose Gas Chromatography coupled with *time-of-flight* Mass Spectrometry (GC-*tof*-MS) [12], [27]–[29] in combination with multivariate analysis, namely Random Forests (RF) [30] and dissimilarity Partial Least Squares Discriminant Analysis (d-PLS-DA) [31], were used to identify large number of VOCs. This database of VOCs was used to search for classifying predictors. To our knowledge this is the first study in which VOCs profiling is used to predict asthma in preschool children.

## Materials and Methods

### Study design and asthma diagnosis

The study population consists of children from the “Asthma DEtection and Monitoring” (ADEM) study (registered at clinicaltrial.gov: NCT 00422747) that started in the Netherlands (Maastricht). The Ethics Committee of Maastricht University approved the study protocol. Moreover all parents gave written, informed consent. The aim of this study is to develop a non-invasive instrument for an early asthma diagnosis by using biomarkers of airway inflammation in exhaled breath. The complete design of the ADEM study including the patient recruitment, primary hypotheses and power calculations has been published before [32]. Briefly, children between the age of 2–3 years old were selected based on respiratory symptoms, with inclusion criterion requiring at least 2–3 episodes of wheezes during child's life (based on parents-completed International Study of Asthma and Allergies in Childhood (ISAAC) questionnaire). Additionally a control group was obtained of children aged 2–3 years without any episodes of wheeze and other recurrent respiratory symptoms during their life (also based on parents-completed ISAAC questionnaire). Exclusion criteria were: mental retardation, cardiac anomalies, congenital malformations, other diseases of the lungs/airways, Crohn's disease or rheumatic arthritis, and the inability to perform lung function measurements or exhaled breath collection. After written informed consent, children and parents were invited for a visit to the lung function laboratory. The lung function assistant and/or research physician further evaluates appropriateness for participation. A questionnaire including information on demographic data, medical history of the child, family history, day-care attendance, housing, prescribed drug therapy, exposure to pets, and passive smoking was completed. In the ADEM study children were followed prospectively until the age of 6 years. In the study in total 252 children were encompassed. From these children 202 individuals with recurrent wheezing symptoms were selected and 50 healthy controls without wheezing episodes until inclusion [33]. At the age of six years children who participated in the study were classified as healthy, transient wheezers or true asthmatic by an experienced pediatric pulmonologist and a computer algorithm based on status at inclusion (healthy control or transient wheezers), symptoms, lung function and used medication. A detailed description of final diagnosis of asthma in ADEM study can be found elsewhere [34]. Note that at age of six years one child, who was at inclusion selected as healthy, was classified as asthmatic. Moreover from the set of 202 children, 6 of them remained unclassified in term of diagnosis.

Over these years each child delivered 3–7 breath samples. Table 1 shows the overview of clinical characteristics and number of breath samples and individuals used in the study. Different measurements of the lung function were performed both at inclusion and age 6 (see Table 1). Significant differences (p<0.05) in lung function (FEV1/FVC at age 6) were only observed between healthy and asthma as well as between healthy and transient wheezers. Eczema was more frequent in transient wheezers and asthmatic children compared to healthy. There were no differences in age or gender between studied groups.

**Table 1:** Clinical characteristics of the children involved in the study subdivided in healthy, transient wheezers and asthma at age 6.

	Healthy	Transient wheezers	Asthma
Total number of breath samples	185	546	343
Number of individuals at inclusion	49	121	76
Number of individuals at age 6	49	121	76
Age of inclusion (mean ± std)	3.3±0. 5	3.3±0.65	3.1±0.7
Male/Female	24/25	62/59	45/31
FEV1* at age 6 (in ml; mean ± std)	1856±2111	1895±2164	1720±1963
FEV1/FVC** at age 6 (mean ± std)	90.5±5.8	88.2±6.9	86.2±7.3
Eczema at inclusion (in %)	22%	34%	46%
Eczema at age 6 (in %)	22%	37%	47%
MicroRint*** at inclusion (in kPa*s*L^−1^)	1.45±0.38	1.45±0.35	1.55±0.37

* Forced expiratory volume in 1 second in spirometry;

** Tiffeneau-Pinelli index: the proportion of a person's forced vital capacity in the first second of expiration;

*** a test to measure airway resistance.

### Sampling and measurements

The subjects involved in the study breathed through a facemask connected to a valve of a resistance-free 1L plastic bag (Tedlar bag, SKC Ltd, Dorset, UK). One hour before sampling, eating and exercise were not allowed. The use of inhaled corticosteroids was stopped four weeks before the measurements except in 7 patients due to severe asthma symptoms. Children participated in the study were sampled randomly without division on healthy controls and children with wheezing. All samples from subjects taking part in this study were collected in the same room in order to prevent the appearance of a background bias. The plastic bags were emptied via pump with constant flow over a stainless-steel two-bed sorption tube, filled with carbograph 1TD/Carbopack X (Markes International, Llantrisant, Wales, UK) within 1 h after collection. The air-tight capped tubes were kept at room temperature until analysis (in average for three weeks). The capped tubes can be kept at room temperature up to six months without significant changes of VOCs profile. The bags were cleaned by filling and empting 2 times with nitrogen and reused for next measurements. In this study 1L of mixed breath (end-tidal and dead space air) was collected. Dead-space air comprises only a small part (30 ml) of the total sample of exhaled air collected and we have shown that the contribution of dead-space air to the total volume of whole breath does not lead to sensitivity issues in measuring VOCs by GC-*tof*-MS [28].

The exhaled air samples were measured by means of GC-*tof*-MS [35]. All collected samples were measured randomly, i.e. the batch of 26 samples a random set of breath samples obtained from healthy controls and children with wheezing. The GC-*tof*-MS method applied here is a non-targeted GC-*tof*-MS method, i.e. no prior identification of the compounds was performed. All chromatographic conditions were optimized by us previously [28] and consequently in consultation with the producer of our instrument and based on common chromatographic experience we chose a column and the temperature programming that were suitable to detect many different classes of volatile compounds and at the same time keep the best possible separation of the compounds at a high sensitivity and a high dynamic range. The detailed parameters of GC-*tof*-MS measurements are listed in Table 2.

**Table 2:** GC-*tof*-MS measurements and used parameters.

Step	Description	Parameters used
1	Desorption of the tubes	10 min under a flow of Helium (50 ml/min); Temp:350°C
2	Injection of the sample onto GC column	Carrier gas: helliom (1.5 ml/min); Temp.: 40°C for 5 minutes then increased by 10°C until 270°C
3	Mass spectrometer scanning	35–350 AMU (5scans/second)

### Data preprocessing

**Figure 1:**
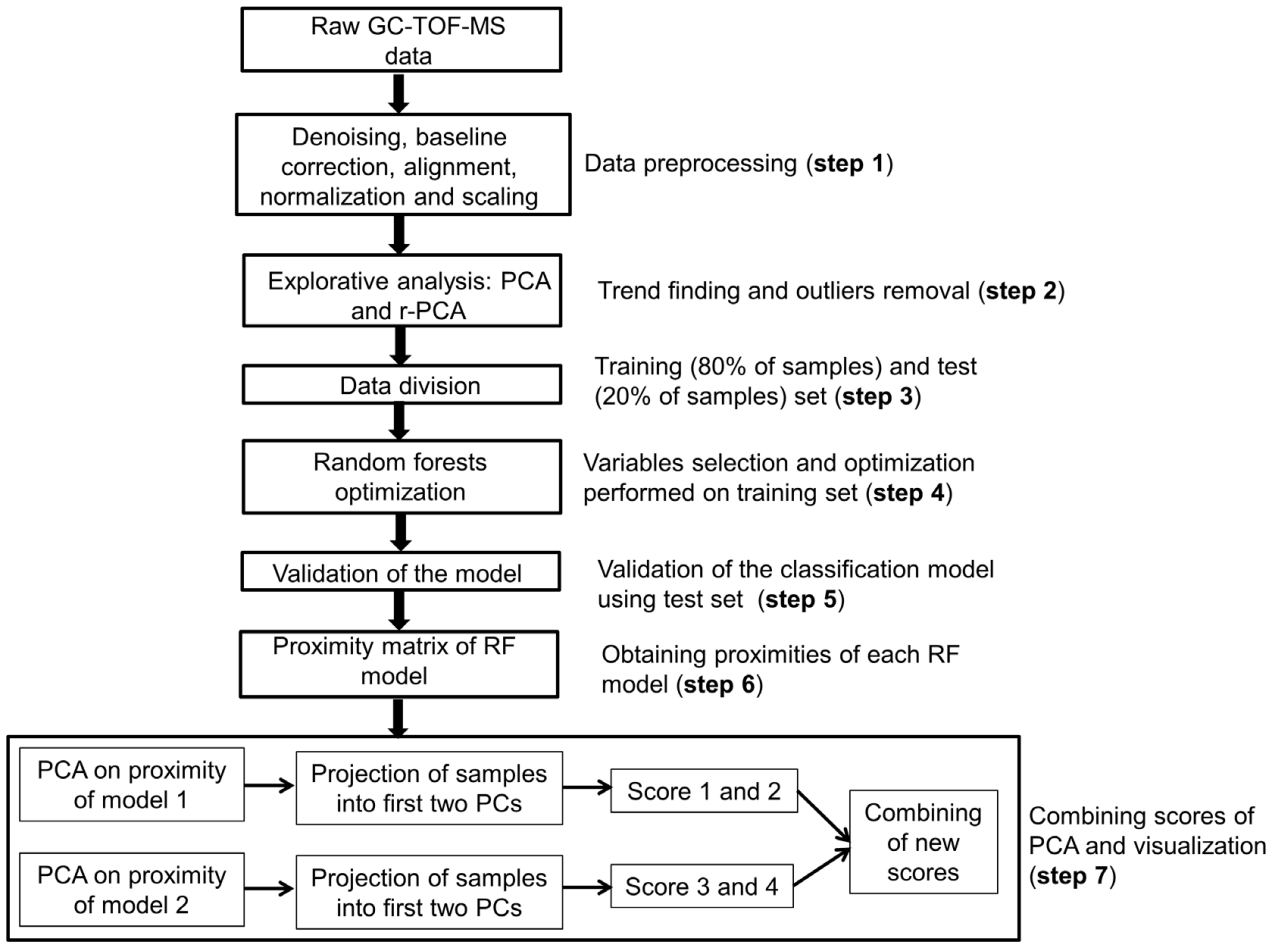
Flowchart of the different steps in data preprocessing and analysis. In step 7 model 1 corresponds to RF analysis of groups healthy and asthma, while model 2 to RF analysis of groups transient wheezers and asthma.

The conceptual flowchart presenting the approach including the data preprocessing and data analysis used in our study is presented in Figure 1. The **first** step in the approach consists of the preprocessing of raw output data from GC-*tof*-MS. This phase is particularly important, because it has influence on all subsequent steps. Data preprocessing techniques usually can improve the quality of the data, thereby helping to make the accuracy and efficiency of the subsequent data analysis process better. All data preprocessing steps were performed in Matlab version R2013a. First of all, the beginning and end of each chromatogram (retention index in minutes either <1.3 or >23 min) were removed because of noisy mass spectra at the beginning of the chromatograms and column bleeding at the end of each run. The next step consists of noise removal and baseline correction (removing chromatographic background). Denoising was performed by means of wavelets transformation (with Daubechies wavelet and two levels of compressions by wavelet toolbox, Matlab 2013a) [36]. In the following step the background was corrected via P-splines with smoothing parameter α of 10^8^ and penalty of 0.1 [37]. In order to apply multivariate analysis the chromatographic drifting due to column ageing, temperature differences and different sample composition has to be corrected. Retention times of all samples were corrected by Correlation Optimized Warping (COW) using segment length of 20 and degree of warping in segment length of 7 [38]. The algorithm for COW is available in [39]. Total ion current (TIC) chromatograms were progressively aligned using segmental linear compression and/or stretching in order to maximize the correlation to a reference chromatogram. For each peak in TIC chromatogram the area under the peak is calculated. Note that these areas are proportional to relative amount of measured compounds. The absolute concentrations of the compounds were not determined. Next, the peak areas and the corresponding mass spectra are compared for all samples. In order to merge the same compounds along all available samples the similarity (based on correlation) between mass spectra is calculated. To make the spectra comparable the final step of preprocessing involved normalization to total area. For Random Forest (RF) [30] models the data were not scaled, while before dissimilarity Partial Least Squares Discriminant Analysis (d-PLS-DA) [31] model rank transformation was applied to the data. RF and d-PLS-DA were performed in Matlab R2013a (Statistics Toolbox) and in-house written function.

The example of four breathograms before and after preprocessing is shown in Figure S1.

### Explorative and supervised analysis

In step **2** of our approach the explorative analysis by means of robust-Principal Component Analysis (r-PCA) [40] and PCA was first used to control the presence of outliers and to find eventual trends and groupings in the data. The strategy for supervised data analysis involved first (step **3**) dividing data into a training set (80% of samples per class) and an independent test set (20% of samples per class) by using the Duplex algorithm [41]. The training set was used for optimization steps (i.e. variable selection and selecting model complexity) and for developing a classification model, here RF [30] and d-PLS-DA [31]. The test set was subsequently used to validate the constructed model. In case of RF model an extra validation is employed using so called out-of-bag (oob) error. For each RF tree one-third of the training samples were left out and not used in the construction of the classification model.

### RF analysis

The VOCs data of our ADEM study contain three main classes: healthy controls, transient wheezing and asthma. In step **4** of our approach, two RF models were constructed, each based on 1000 trees. First the classification algorithm was applied on data containing all samples belonging to healthy controls and asthma to find VOCs related to abnormal status occurring in the lungs. The second model was constructed on data containing all transient wheezers and asthmatic children with the purpose to find compounds related solely to asthma. Compound importance is found by permuting the values of each variable in the oob cases and predicting the values of these samples [30]. By randomly permuting the values in the predictor variable “i”, the association with the class vector is lost. When the prediction accuracy for the cases in oob decreases significantly in comparison with non-permuted variables values, it indicates a strong relation of the predictor variable “i” with the response (i.e. classes). The difference in prediction accuracy before and after permuting the values of variable “i”, averaged over all trees, is a measure of variable importance. The higher the number, the more important the predictor variable “i” is.

Each model was validated in step **5** by means of oob error estimate and separately by the independent test set. It ought to be mentioned that validation is a crucial step of any supervised algorithm [42]–[44]. A classification model is considered statistically valid if it shows good prediction ability on an independent test set. Two types of predictions for independent test set were calculated. The first one did not account for multiple measurements per subject. The second type called subject-level classification rate, was obtained on individual level by majority votes. The subject classification was calculated as the class with the maximum number of votes across all measurements for the subject. The tie was treated as undefined class. In step **6** proximities for each RF model were obtained. The proximities originally formed an n×n matrix (where n is a number of samples). After each tree was constructed, all of the data were put down the tree. If two samples ended in the same terminal node their proximity was increased by one. The final proximity was normalized by dividing by the number of constructed trees. Proximity can be compared to similarity matrix, thus the higher the number, the more similar the samples are. In other words it represents similarity scores computed between two samples. Combination of the two classification models (**step 7** in Figure 1) allows visualization of the differences between healthy controls, transient wheezers and asthmatics. For that purpose proximities of both RF models (healthy vs. asthma and transient wheezer vs. asthma) were used and PCA was performed on them. By using loadings obtained from each PCA model it is possible to project the remaining samples (i.e. the ones not used in the RF model) into the PCA score plot (here the first two PCs). In this way for each sample new scores were obtained [45]. By combining these new scores it is then possible to visualize relation between all samples used in the study (**step 7** in Figure 1).

### D-PLS-DA analysis of transient wheezers and asthmatic children at the early age

The main goal of the paper is to demonstrate the feasibility of VOCs profiling to differentiate early stage of asthma from transient wheeze in preschool children. Therefore, in order to represent only the differences in VOCs profile between asthma and transient wheeze at the day of inclusion and to corroborate the results found by RF, d-PLS-DA was constructed using compounds selected by the previous two RF models. D-PLS-DA is a variation of PLS-DA, where the original data are first transformed into dissimilarity measure. In d-PLS-DA a linear model is then constructed according to equation 1:
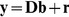
(1)where, **D** is a dissimilarity matrix, **y** is a vector of group memberships, **b** is a vector of regression coefficient, and **r** is a vector of model residuals.

The Euclidean distance was selected as the dissimilarity measure [31]. A crucial step in a PLS model construction is the selection of the optimal number of latent variables (LVs) (so called model complexity). Usually, this is done by using the cross-validation (CV) procedure. Here leave-one-out CV was applied. In this approach during an iterative process one sample from training set is left out at each step, while the remaining samples are then used to build d-PLS-DA models using different number of LVs. For each sample that is removed, the average prediction errors are obtained. They express the performance of the classification model with respect to the number of LVs. The optimal number of LVs was selected based on the minimal error of the root mean square error of cross-validation (RMSECV).

## Results

### Data

In total 1124 GC-MS spectra were measured of which 50 were removed due to poor quality of the recorded spectra. The final 1074 breath-o-grams were preprocessed as described in Materials and Methods and the resulting data matrix contained 3256 variables (peaks representing individual VOCs). The data set has a lot of zeros, since not each compound is present in each breath sample (typically 300–500 compounds are measured per breath sample). To reduce the number of zeros present, the following procedure was applied, which will be referred here as the “20% rule”. A variable is kept if it has a nonzero value for at least 20% of samples in at least one of the experimental groups. After the 20% rule, the final data matrix contains 527 variables.

### Explorative and supervised analysis

**Figure 2:**
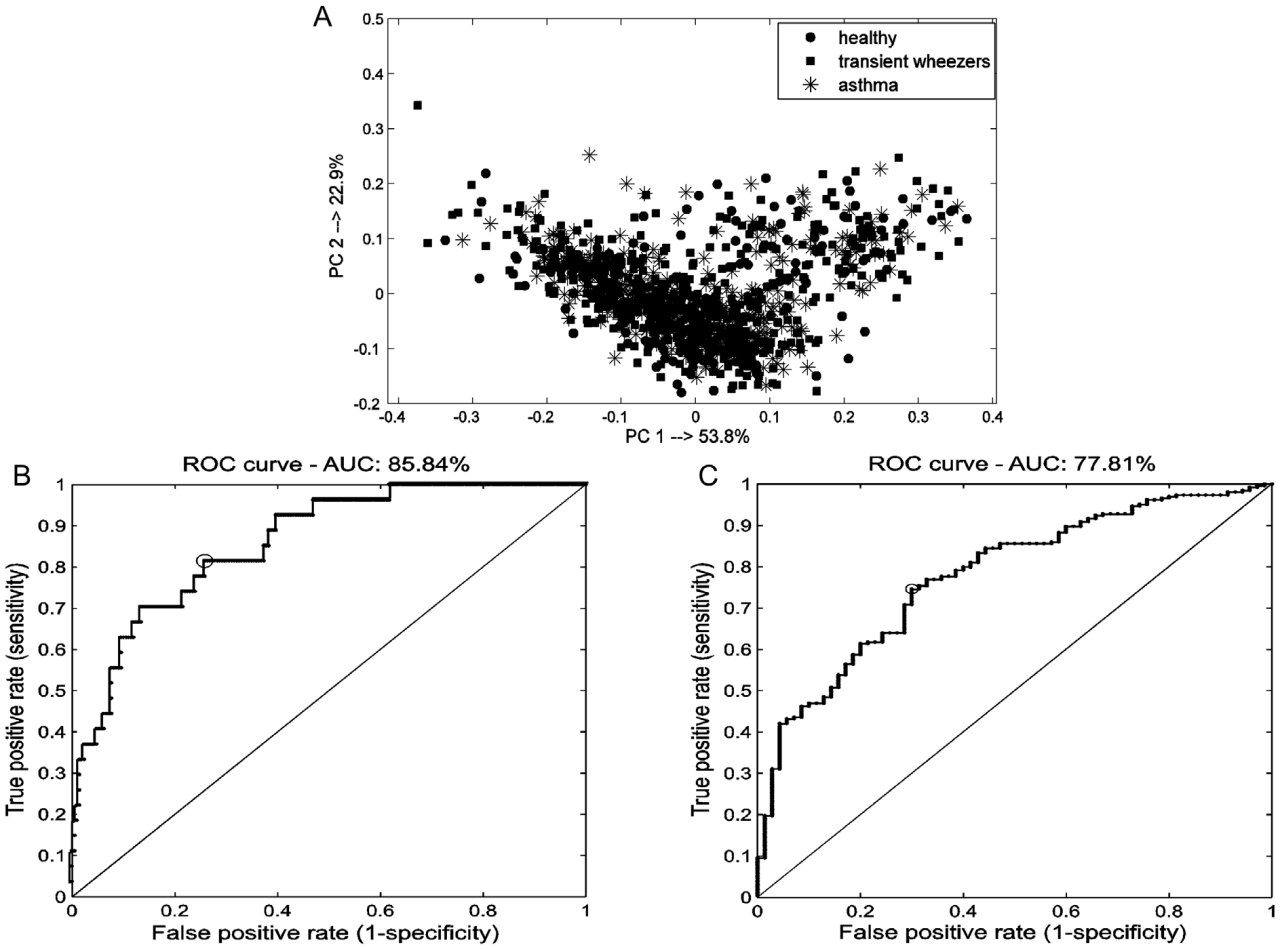
The outcomes of PCA and ROC analysis. (**A**) PCA score plot of 1074 breath-o-grams performed on 527 VOCs excreted in breath samples obtained from healthy, transient wheezing and asthmatic children. There is no clear grouping visible; (**B**) ROC curve for independent test set of the RF model obtained for groups healthy and asthma using 12 VOCs. The area under the curve is 85.8%; the sensitivity and specificity for the optimal cut-off of 0.52 (indicated as circle in the figure) are respectively equal to 81.5% and 74.2% (**C**) ROC curve for independent test set of the RF model obtained for groups transient wheezers and asthma using 12 VOCs. The area under the curve is 77.8%; the sensitivity and specificity for the optimal cut-off of 0.51 (indicated as circle in the figure) are respectively equal to 74.6% and 70%.

After pre-processing, the explorative analysis was performed by means of r-PCA and PCA. Since each individual delivered several breath samples (from 3 to 7), inter-individual variation may obscure the information of interest. The 1074 breathograms are not independent measurements. Having multiple measurements for each individual was addressed in selecting the training and test set samples. The test set contained always all measurements belonging to the same subject. Thus the RF classification model was never trained on part of measurements of the same subject and test on the remaining measurements of the same subject. Moreover, to diminish the influence of inter-individual variation centering per individual was performed. This allows converting all the relative compound concentrations to fluctuations around zero for each individual instead of around the population mean. In other words biological mean for each individual is set to zero. Initially, r-PCA was applied to the mean centered breath-o-grams of 1074 samples, to check for outliers. No outliers were detected. As can been seen in the PCA score plot of all breath-o-grams no clear groupings are observed since the samples from all three groups overlap (Figure 2A). This indicates that the majority of the variance in the data does not correspond to the available groups. It is important to mention that further PC's did not show groupings either. This observation justifies the use of supervised analysis.

### RF analysis

In our study we have three groups (healthy, transient wheezing, true asthma) and although RF can be used for more than two classes, we decided to split the classification into two separate models. This approach gives simpler interpretations of the outcomes, since it focuses on one particular aspect at a time. In the RF model of healthy vs. asthma the studied aspect is for instance inflammation in the lungs, which is a common feature of asthma and transient wheeze. In the second RF model, i.e. transient wheezers vs. asthma, the features are directly related to either asthma or wheezing.

The comparison between healthy and asthma has been performed in order to find the compounds that represent deviant processes in the lungs. This model leads to a set of 12 compounds that allows differentiating healthy and asthma. The overall prediction for the independent test set was 77.1% with correct classification rate for asthma and healthy equal to 74.6% and 81.5%, respectively. The subject-level correct prediction for asthma and healthy was equal to 73.2% and 82.3%, respectively, In Figure 2B the Receiver Operating Characteristic (ROC) curve is shown for the independent test set. This ROC curve and the area under the curve (85.8%) indicate that healthy individuals and asthma group are quite well separated and the RF model has a good model performance.

In the second RF model we compared children suffering from asthma and transient wheezers. This model had relatively good performance with the overall correct classification for the test set of 70.9%. The correct prediction for the test set for asthma and transient wheezes was correspondingly equal to 71.4% and 70.1%. The subject-level correct classification for transient wheezers and asthma reached 73.3% and 72.1%, respectively. The ROC curve for the independent test set is shown in Figure 2C. The RF model yields 12 compounds capable to discriminate asthma and transient wheezers.

### Combining PCA score plots of proximities

**Figure 3:**
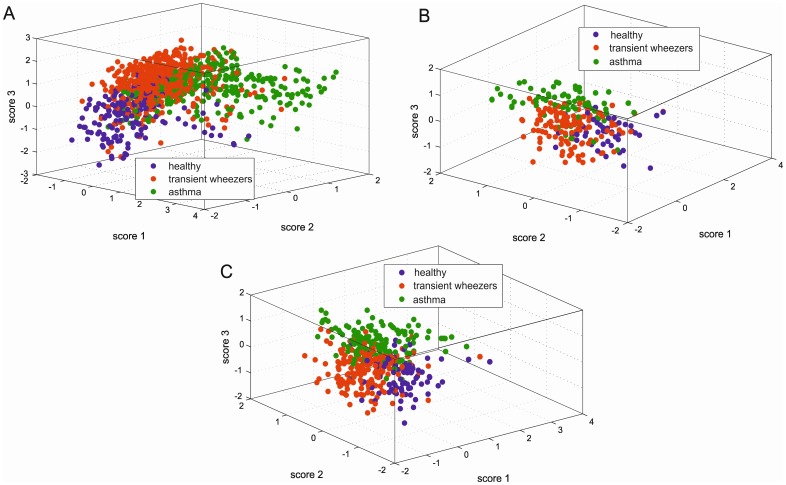
Projection into score 1, score 2 and score 3 delivered from PCA on proximities of RF model 2 (transient wheeze vs. asthma) and model 1 (healthy vs. asthma). (**A**) of all 1074 breath samples obtained from children collected over time starting at age 2 and finishing at age 6 years old; (**B**) of breath samples collected at early age (i.e. inclusion at age 2–4 years); (**C**) of breath samples obtained at day of final diagnosis (i.e. age 6 years old). Each breath sample is color-coded accordingly to group's membership: healthy, transient wheeze and asthma.

As indicated in the Materials and Methods section, proximities obtained from RF models give the possibility to study groupings and trends in the data. The higher the proximity is between two samples, the greater the similarity. For RF model 1 (healthy vs. asthma) and RF model 2 (transient wheezers vs. asthma) proximities were calculated and next PCA was performed on these proximities matrices. The projection of remaining samples (i.e. not used in RF model) into PCA plane enables creating for each sample a set of new scores. Since the two first PCs describe the largest amount of variance (here around 50%), for each sample 4 new scores are created (two scores from each PCA model). Projecting the samples into PCA performed on proximities of RF models for groups transient wheezers and asthma resulted in score 1 and score 2, while for groups healthy and asthma in score 3 and score 4. The combination of score 1, score 2 and score 3 represents the relation between the groups and their separation. The corresponding graph for all 1074 breath samples is shown in Figure 3A. As can be seen the three studied groups are separated using these three scores. The overlaps observed between samples belonging to healthy and transient wheezers as well as between asthma and transient wheezers indicate the partial similarity in VOCs profile of these groups. Interestingly there is clear separation between samples related to healthy and asthma groups. It ought to be mentioned that Figure 3A shows all breath samples collected from children at 2 to 4 years old and followed prospectively until the age of 6 years. However, it is possible to focus only on breath samples collected from children at the early age (i.e. inclusion at age 2–4 years) and at day of definitive diagnosis (i.e. age 6 years), and to visualize the possible separation and relation between groups. Figure 3B presents only the breath samples collected from children at the early age (i.e. inclusion at age 2–4 years old). As can be observed the separation between asthma and transient wheezers groups is achieved. The fractional overlap between the groups of healthy and transient wheezers indicates similarities in VOCs profile at early age of 2–4 years. It is worthwhile to point out that the asthmatic and transient wheeze samples are well separated mostly along score 3. In Figure 3C the visualization of individuals sampled at age 6 years (day of final diagnosis) is demonstrated. Similarly to the previous two figures the best separation exists between asthma and healthy groups. Nevertheless, the asthmatic and transient wheezing samples create two separate clouds of points.

### D-PLS-DA model of asthmatic and transient wheezers at inclusion

**Figure 4:**
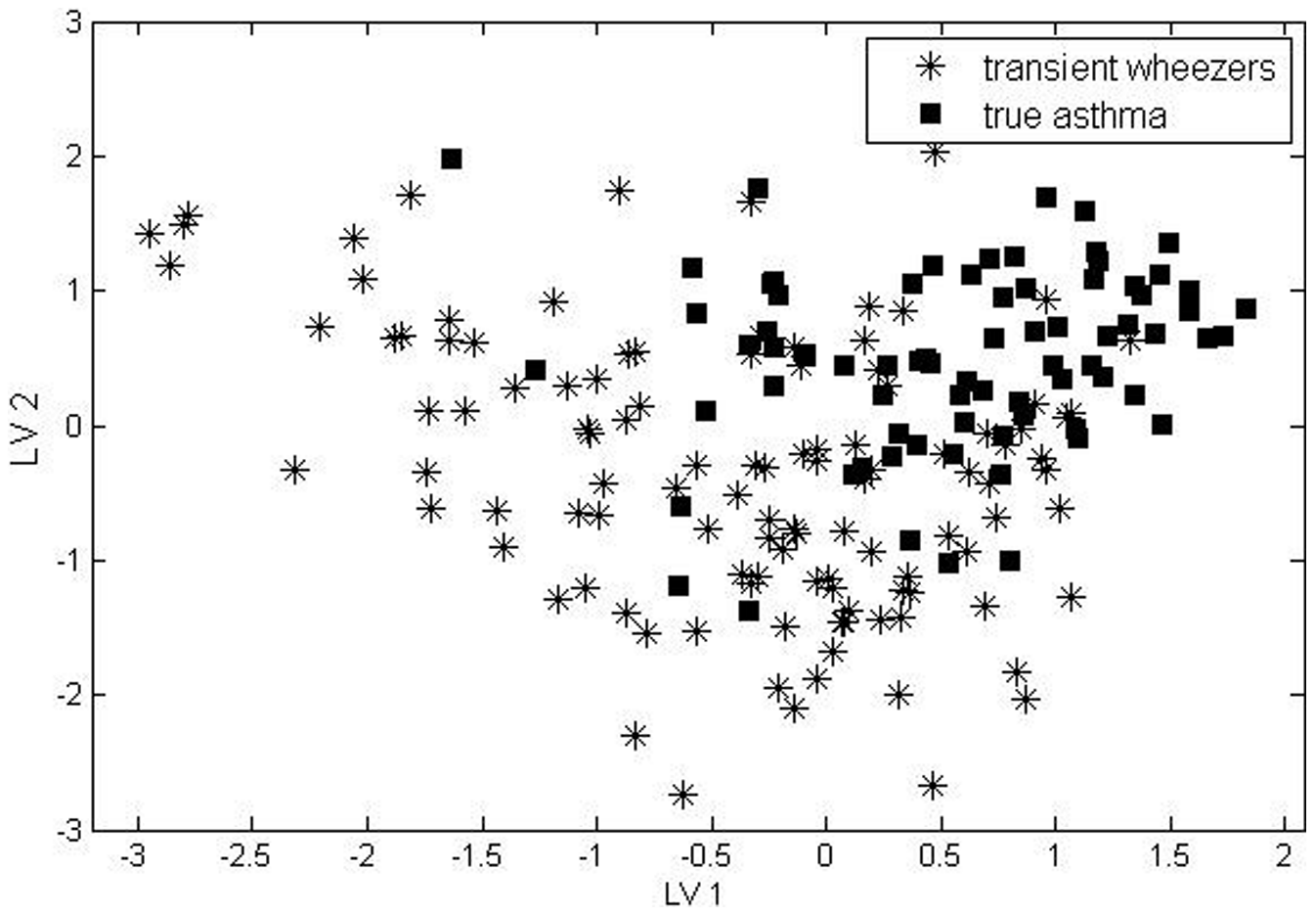
d-PLS-DA score plot, projection of objects into the space of two PLS latent variables of data containing children with transient wheeze and asthma at the early age (inclusion age 2–4 years old).

To further confirm the outcomes obtained from RF models we used d-PLS-DA as a corroborative technique to demonstrate the differences between asthma group and transient wheezers group at inclusion (i.e. at age 2–4 years). In this model we used the significant compounds found by RF model 1 (healthy vs. asthma) and RF model 2 (transient wheezers vs. asthma). In total 17 compounds were used. The optimal complexity of the model found by RMSECV is 2 LVs. The resulting d-PLS-DA score plot is shown in Figure 4. As can be seen the groups are not perfectly separated. Interestingly, the transient wheezers group shows more variation than the asthma group, since the spread of the samples is large. This indicates that the group of transient wheezing children is more heterogeneous then the asthma group. The d-PLS-DA model was validated by using test set. The overall accuracy of the d-PLS-DA model for the test set is 80%. More importantly this model has good prediction ability for both groups with correct classification rate of 73.3% and 86.7% for asthmatic and transient wheezers individuals, respectively. This means that limited number of compounds in exhaled breath sampling at the early age (2–4 years old) can predict with high accuracy whether a wheezing child is a transient wheeze or develop true asthma at age 6.

### Compound identifications

The chemical identification of the 17 VOCs was done by means of spectrum recognition using the The National Institute of Standards and Technology library in combination with spectrum interpretation by an experienced mass-spectrometrist and identification based on retention times of compounds. From a set of 17 VOCs it was possible to chemically identify 14 of them while three remains unknown. These 3 compounds could not be identified due to insufficient mass spectrum, overlap in the retention time or absence of mass spectrum in the library. Table 3 shows the list of 14 identified VOCs and their relative concentrations change in breath samples obtained from asthmatic children at day of inclusion. Up or down regulation of relative VOC concentration is indicated as (+) or (−), respectively, with reference to transient wheeze. As can be observed the discriminatory compounds belong mostly to different alkanes.

**Table 3:** A list of 14 chemically identified discriminatory VOCs as measured at early age.

	Group
VOCs	Early asthma
Acetone	(−)
2,4-dimethylpentane	(+)
2,4-dimethylheptane	(+)
2,2,4-trimethylheptane	(−)
1-methyl-4-(1-methylethenyl) Cyclohexen	(−)
2,3,6-trimethyloctane	(−)
2-undecenal	(+)
Biphenyl	(−)
2-ethenylnaphtalene	(−)
2,6,10-trimethyldodecane	(−)
Octane	(+)
2-methylpentane	(+)
2,4-dimethylheptane	(+)
2-methylhexane	(+)

(−) indicates decrease in VOC relative concentration, while (+) increase in asthmatic children with reference to children with transient wheeze as diagnosed at age 6 years.

## Discussion

The analysis of the total array of VOCs in exhaled breath has gained popularity over the last few years as a diagnostic and monitoring tool in medical settings. Hundreds of different VOCs are present in human breath and their relative concentrations may change in response to abnormal physiological processes in the body. VOCs are a various group of carbon-based chemicals that are volatile at ambient temperature which are formed in the body, for instance as a result of lipid peroxidation, protein metabolism or cholesterol biosynthesis [5]. Due to their low solubility in blood VOCs are easily excreted into breath after entering the lungs.

In this paper we demonstrated the use of VOCs in exhaled breath may lead to development of alternative ways of monitoring, diagnosing and studying health status. The detection of exhaled VOCs by GC-*tof*-MS produces huge datasets of biological variables. The analysis of such data requires the use of machine learning methods [46]. Based on previous experiences and preliminary results (not shown) we decided to use RF and d-PLS-DA as classification methods in discriminating transient wheezers and asthmatic children. In contrast, a linear method (PLS-DA) failed. The two classification algorithms are suitable for dealing with different degrees of nonlinear problems. Here, RF was firstly used to select significant variables and next for visualizing the relation/differences between studied classes. D-PLS-DA, a method based on a completely different paradigm, was applied to corroborate the results obtained by RF and thus ensured the certainty of the selected variables and their discriminatory power.

One aspect of the current study is the presence of cluster-correlated data (i.e. multiple measurements are available for each individual). This might cause an underestimation of the classification error or overstating the significance of the effect interest[47]. In case of RF it has been shown [48], [49], that it is justified not to account for cluster-correlated measurements if two precautions are respected. Firstly, the outcomes should be validated on an independent test set, which is not used in the building of the RF classification model. Secondly, it is required to use an additional post-processing step that involves a prediction performance to obtain subject-level classification [49]. This was also verified in our case (data not shown). Both requirements were met in our current study. The performance of RF was relatively similar with or without taking into account cluster correlated measurements. In average the overall correct classification for approach accounting for cluster correlated measurement was equal, for 100 iterations, to 77.8±3.2% and 71.7±2.3%. The approach used in this study led to averaged correct classification rate, for 100 iterations, 76.9±1.3%and 71.5±1.5%. Clearly, the results are comparable. However, one can see that the standard deviations are larger when accounting for cluster correlated measurements. The similarity of these results might be explained by bootstrap aggregating, i.e. sampling with replacement used by RF algorithm. Within bootstrap aggregating, a subject sampled with replacement from a population can be similar to another sample from the same population (e.g. repeated measurement of the same subject). Therefore, sampling very similar repeated measurements has almost the same effect as sampling with replacement. In case of low correlation between the repeated measurements, the replicates are nearly independent thereby eliminating the cluster correlation issue.

Obviously, VOC data from GC-*tof*-MS are numerically very complex and contain many irrelevant and redundant features. Therefore we applied variable selection to make sure that accurate information extracted from the data can be used in the final classification model. Using only a limited number of compounds we were able to distinguish healthy, asthmatic and transient wheezing individuals with very good prediction in an independent test set. More importantly we were able to distinguish between preschool asthma and transient wheezers at an early age, i.e. inclusion age was 2–4 years. The overall correct classification rate was equal to 80%. Although imperfect such a result based on the analysis of VOCs in exhaled breath is a very attractive alternative for assessing asthma in children at that age. Current methods are invasive and very often not possible to perform in preschool children, because these tests require active cooperation from the examined person. Collection of breath samples is safe, non-invasive and easy to perform even in infants.

A limited number of 17 VOCs was equipped to classify children with asthma and transient wheeze at the onset of the disease. The same set of VOCs was also capable of differentiating all available samples (see Figure 3A) as well as children with asthma and transient wheeze at age of final diagnosis, i.e. age of 6 years (see Figure 3C). The boxplots of 5 most discriminatory VOCs (2-methylhexane, octane, 2,3,6-trimethyloctane, acetone and 2,6,10-trimethyldodecane) are included in the supplementary material (Figure S2). The variation of VOCs at age of inclusion (2–4 years) and at age of final diagnosis (i.e. age of 6 years) for transient wheezers and asthmatic children is for most of the discriminatory compounds comparable. The variability of octane and 2,3,6-trimethyloctane is higher in asthmatic group at age of 6 years in comparison to asthmatic group at age of inclusion (Figure S2 B and C). The set of discriminatory compounds was not unique to asthmatic children but was also observed in transient wheezes in higher or lower concentration. The set of discriminatory compounds shown in Table 3 are mostly hydrocarbons. In case of asthma, airway inflammation plays a crucial role in the pathophysiology of the disease. Because of the imbalance between oxidants and antioxidants a degradation of polyunsaturated fatty acids, found in cellular membranes, takes place during the airway inflammation [4], [5]. This imbalance is caused by the continuous production of reactive oxygen species (ROS), which is triggered by an influx of leukocytes characteristics for lung inflammation. During this process hydrocarbons are created by lipid peroxidation of ω-3 and ω-6 polyunsaturated fatty acids. The alkanes found to be discriminatory in our study belong to longer chain alkanes which might be more specific to asthma. Previously similar types of alkanes were found in asthma [7], [8], [29], [50], [51]. It has been proposed that the group of compounds of C7 to C12 alkanes and their methylated derivatives are indicative for lung cancer [52] but also require further exploration as potential biomarkers for asthma [8]. Our findings are in line with previous ones [7], [50] and may be explained by the complex equilibrium between formation and removal of VOCs in the body. Besides oxidative stress assumption, the changes in VOCs relative concentration can be explained by differences in gas exchanges over the blood- lung barrier between asthma-affected and transient wheezers-affected lungs. This consequently might influence the alterations in relative VOCs level in exhaled breath. Acetone was one of the found discriminatory compounds. This is the most abundant VOC in exhaled breath. Therefore, the changes in acetone level may be due to artifact caused by normalization to total area. Consequently, another type of normalization, namely probabilistic quotient normalization, was applied and the results compared. No differences were observed. Thus the changes in acetone level are the results of biological processes.

Breath analysis has been used in various studies and several VOCs have been reported as discriminatory for asthma, COPD and CF [7], [11]. However in most of the studies, due to for instance limited number of subjects, the outcomes were not completely validated, i.e. only cross-validation was used. On contrary, in our study we used an external test set (so called independent test set). This is a higher level of validation and therefore gives stronger certainty to the findings. Nevertheless, further validation in new cohorts is recommendable before the use of these discriminatory compounds in the clinic.

## Concluding Remarks

In conclusion, we demonstrated that a VOCs profile in exhaled breath was able to discriminate healthy, transient wheezing and asthmatic children. The VOCs profile allowed distinguishing children with transient wheeze from true asthmatic. Asthma is an inflammatory disease of the lung with oxidative stress in the cells lining bronchi which leads to lipid peroxidation. This process seems to play a crucial role in the production of differentiating VOCs in exhaled breath of asthmatic subjects. However, it cannot be excluded that the VOCs come from other pathways. Further research is needed to establish the etiological pathways. Despite the potential use of VOCs in clinical practice many aspects need to be standardized, such as sampling procedures and measurements. The development of international recommendations for standardized procedures in the analysis of VOCs of exhaled breath would improve inter-laboratory comparisons.

## Supporting Information

Figure S1 **The example of 4 breathograms; (A) before data preprocessing and (B) after applying denoising, baseline correction and alignment.**
(TIF)

Figure S2 **The boxplots of the most discriminatory compounds for transient wheezers and asthmatic children at age of inclusion (age 2–4) and age of final diagnostic (age 6): (A) 2-methylhexane; (B)octane; (C) 2,3,6-trimethyloctane; (D) acetone; (E) 2,6,10-trimethyldodecan.**
(TIF)
