# Correction: Profiling of Volatile Organic Compounds in Exhaled Breath As a Strategy to Find Early Predictive Signatures of Asthma in Children

**DOI:** 10.1371/journal.pone.0105447

**Published:** 2014-08-05

**Authors:** The PLOS ONE Staff

There is an error in Table 3. The entry for 2,4-dimethylheptane is duplicated and the entry for 4-methyl-2-pentene is missing. Please see the correct Table 3 here.

**Table 3:** A list of 14 chemically identified discriminatory VOCs as measured at early age.

VOCs	Group Early Asthma
Acetone	(-)
2,4-dimethylpentane	(+)
2,4-dimethylheptane	(+)
2,2,4-trimethylheptane	(-)
1-methyl-4-(1-methylethenyl) Cyclohexen	(-)
2,3,6-trimethyloctane	(-)
2-undecenal	(+)
Biphenyl	(-)
2-ethenylnaphtalene	(-)
2,6,10-trimethyldodecane	(-)
Octane	(+)
2-methylpentane	(+)
4-methyl-2-pentene	(+)
2-methylhexane	(+)
