# Supplementary figures and images for: Profiling of Volatile Organic Compounds in Exhaled Breath As a Strategy to Find Early Predictive Signatures of Asthma in Children
Source: PLoS One. 2014 Apr 21;9(4):e95668. doi: 10.1371/journal.pone.0095668 (PMC3994075; doi:10.1371/journal.pone.0095668)

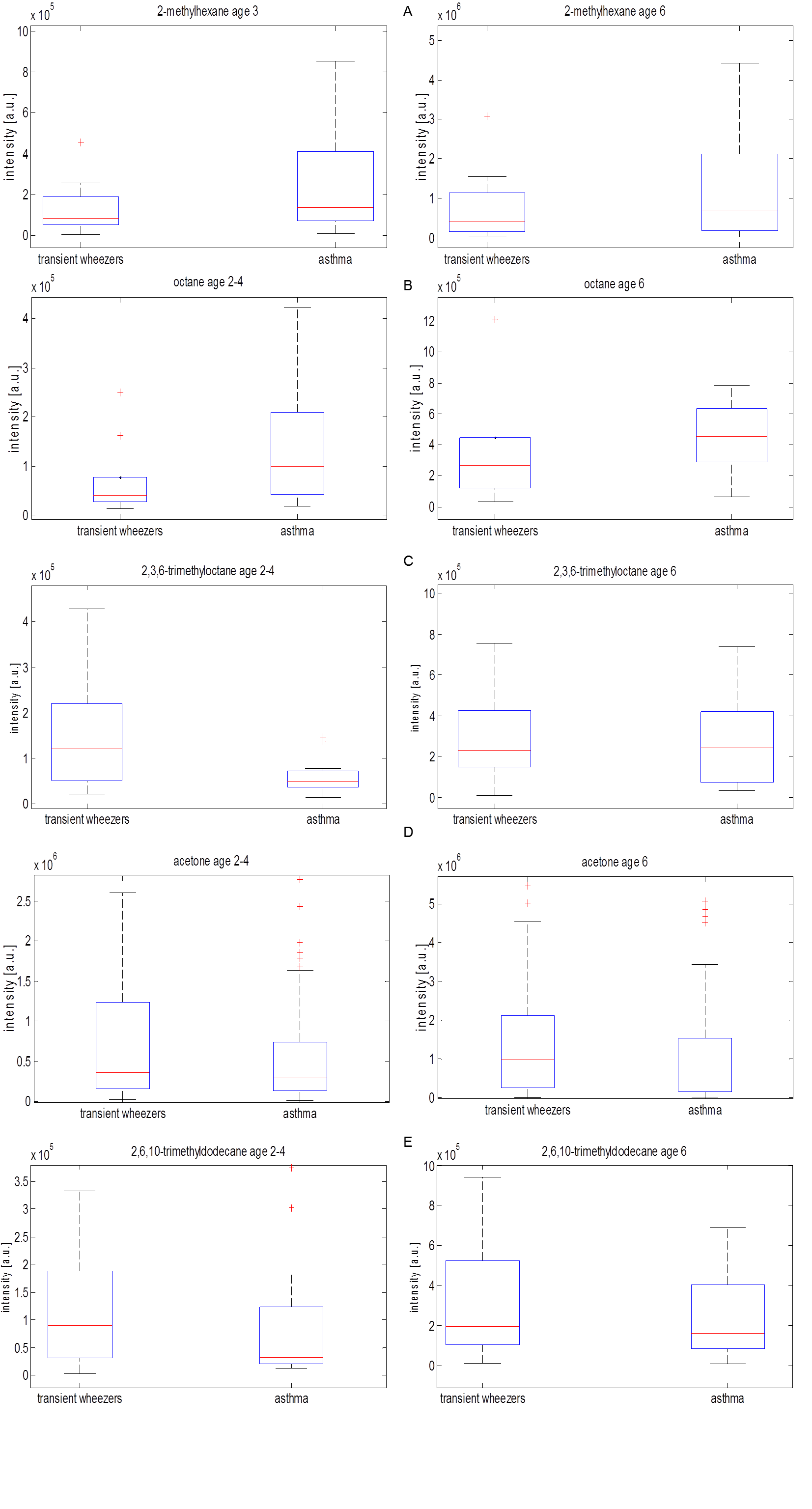

Supplement: Figure S2 — The boxplots of the most discriminatory compounds for transient wheezers and asthmatic children at age of inclusion (age 2–4) and age of final diagnostic (age 6): (A) 2-methylhexane; (B)octane; (C) 2,3,6-trimethyloctane; (D) acetone; (E) 2,6,10-trimethyldodecan. (TIF) [file pone.0095668.s002.tif]
